# Remembering the future; prospective memory across the autistic adult’s life span

**DOI:** 10.1177/13623613231225489

**Published:** 2024-01-19

**Authors:** Annabeth P Groenman, Carolien Torenvliet, Tulsi A Radhoe, Joost A Agelink van Rentergem, Wikke van der Putten, Mareike Altgassen, Hilde M Geurts

**Affiliations:** 1 University of Amsterdam, The Netherlands; 2 Leo Kannerhuis (Youz/Parnassiagroep), The Netherlands; 3 Johannes Gutenberg University Mainz, Germany

**Keywords:** aging, autism, prospective memory

## Abstract

Prospective memory helps us to remember to perform tasks in the future. Prospective memory can be either time or event based. The goal of this study was to determine time- and event-based prospective memory in autistic adults across the life span. Autistic (*n* = 82) and non-autistic (*n* = 111) adults, aged between 30 and 86 years, performed the lab-based Amsterdam Breakfast Task, and several naturalistic prospective memory tasks. Preregistered analyses (AsPredicted #34249) were performed using classical frequentist as well as Bayesian statistics. On none of the prospective memory tasks, group differences were observed. Our results show no effect of age on naturalistic tasks, but age did affect our lab-based measure, indicative of the age paradox often described in non-autistic adults. Moreover, we found evidence for a parallel age-related effect of lab-based and naturalistic prospective memory in autistic and non-autistic individuals.

**Lay abstract:**

*What is already known*: Prospective memory is an important function for daily living. It is the cognitive function that helps you remember that you are meeting your friend for coffee at 2 pm tomorrow, or that you need to take your vitamins after breakfast. This cognitive function is particularly important in autistic adults, but how prospective memory is associated with increasing age, we currently do not know.

*What this paper adds*: Although performance on experimental tasks that measure prospective memory decreases with age, this pattern is similar in autistic and non-autistic adults. No age effects were found for tasks that were performed outside the lab. Autistic adults and non-autistic adults perform similarly on prospective memory, and this performance remains similar when autistic and non-autistic adults age.

*Implications for practice, research, or policy*: While our results show that prospective memory decreased with increasing age, our results do point to parallel development of prospective memory in autistic and non-autistic adults. This finding serves as a reassurance for those individuals concerned that older autistic individuals might show quicker cognitive decline.

Cognitive aging in autism has proved an interesting conundrum. While in typical aging, mostly a decline of cognitive functions has been reported, differential patterns of cognitive functioning have been described in autism (Davids et al., 2016; Geurts et al., 2020; Lever & Geurts, 2016; Pagni et al., 2022; Torenvliet et al., 2022, 2023; Tse et al., 2019). In aging, prospective memory (PM) is an essential cognitive function for independent daily living. PM helps us to remember to perform tasks at the right moment in the future. Think of remembering to take your vitamins after breakfast or remembering to go to your dentist at 1:30 pm. With increasing age, the ability to successfully perform these tasks decreases, which might affect individuals’ ability to live independently (Hering et al., 2018; Sheppard et al., 2020). As higher rates and earlier onset of neurological disorders, including those related to cognitive disorders (i.e. dementia, Parkinson disease), have been reported for autistic adults (e.g. Croen et al., 2015; Geurts et al., 2022; Hand et al., 2020; Vivanti et al., 2021), it is of importance to determine how cognition is affected by age in autistic adults. However, how and which aspects of PM are influenced by age in autistic adults remains unknown.

The literature concerning PM distinguishes between event-based PM (EBPM) and time-based PM (TBPM). EBPM is the self-directed execution of an action when a certain external event occurs, while TBPM is the self-directed execution of a delayed intention at a specific time. While both have to be self-initiated at a certain moment, the external cue for EBPM may prompt rather automatic retrieval of the intended action, whereas TBPM tasks include no external cue and require the individual to monitor the elapsing time in order not to miss the target moment. Therefore, TBPM tasks may require more planning, and intention forming, and be more reliant on executive functioning than EBPM tasks. Indeed, generally it is found that individuals perform less well on TBPM than on EBPM.

Autistic individuals also seem to perform worse on TBPM tasks than on EBPM (Landsiedel et al., 2017; Sheppard et al., 2018). Moreover, systematic reviews indicate that autistic individuals perform less well both on TBPM and on EBPM than non-autistic individuals (Landsiedel et al., 2017; Sheppard et al., 2018). Although the findings of lower performance on time-based tasks in autistic adults seem very robust, results for EBPM are less consistent. For EBPM, only 50% of studies report a lower performance in autistic individuals (Sheppard et al., 2018). Nevertheless, some studies in adults do report group differences on EBPM (Altgassen et al., 2012; Dehnavi & Khan, 2023). Thus, we expect that autistic individuals show worse performance compared to non-autistic individuals in TBPM, but not EBPM tasks.

Adding to the inconsistent findings regarding EBPM is that research is still predominantly performed in children, and the studies that are performed in adulthood have been performed in a wide age range. Moreover, the studies included in reviews (Landsiedel et al., 2017; Sheppard et al., 2018) only include participants under 60 years of age. In typical aging, PM performance over the lifetime can be described as an inverted U, that is, performance reaches a peak around the age of 20 years and slowly decreases with increasing age (Zuber & Kliegel, 2020). However, performance seems to plummet after the age of 60 years (Uttl, 2008; Zuber & Kliegel, 2020). This suggests that a possible age-related decline in PM performance is not captured in current literature on PM in autism, underlining the importance of including older adults in studies into PM in autism.

As TBPM tasks are hypothesized to be more resource demanding than EBPM, it is expected that TBPM tasks typically show a larger age-related decline than EBPM tasks. Studies on PM in older autistic individuals are currently non-existent. However, some cognitive functions necessary for PM, that is, planning, response inhibition, working memory, and retrospective memory, are thought to follow a parallel development (i.e. decrease in a similar manner with age) in autistic compared to non-autistic participants (Davids et al., 2016; Lever & Geurts, 2016; Torenvliet et al., 2022, 2023; Tse et al., 2019). TBPM, which is more dependent on these executive functions, is thought to have a steeper decrease with increasing age compared to EBPM. Therefore, we hypothesize, that because cognitive functions necessary for PM follow a parallel development with non-autistic individuals, PM would follow a similar pattern in autistic individuals as in comparisons.

An interesting paradox has been described in typical aging concerning lab-based versus naturalistic PM tasks. The age-PM paradox describes that younger individuals appear to outperform older individuals on lab-based PM tasks (Henry et al., 2004; Rendell & Craik, 2000), but older adults show superior performance in naturalistic PM tasks (Henry et al., 2004; Schnitzspahn et al., 2020; Uttl, 2008). Lab-based tasks often consist of computerized tasks with an ongoing activity, that has to be interrupted to initiate an action at a certain event (e.g. a red square appearing in EBPM) or a certain time (e.g. every 10 s, in TBPM). Naturalistic PM tasks often entail activities that occur in everyday life, such as posting a letter on a specific day. Naturalistic tasks are thought to be better at capturing difficulties experienced in daily life. Several attempts have been made to explain the age-PM paradox (e.g. more experience with time management, more efficient reminder use, higher commitment to perform naturalistic tasks, more efficient use of cues from the environment, more knowledge of one’s own memory failure (Henry et al., 2004; Phillips et al., 2008)), but the exact nature of this phenomenon remains unclear, and has only been examined in autism once in a small sample (Roestorf, 2018). Here, it was found that older autistic individuals outperform younger autistic individuals on naturalistic tasks. However, this positive association between age and performance was found not only for naturalistic tasks but also for lab-based EBPM. This contradicts the age-PM paradox, possibly indicating that the age-PM paradox is not found in those with autism.

In the current preregistered study (AsPredicted #34249), we will examine aging patterns of PM in autism. We will use the Amsterdam Breakfast Task, an adaptation of the Dresden Breakfast Task (Altgassen et al., 2012), to examine whether patterns of TBPM and EBPM mimic those found in typical aging.

We hypothesize the following:

*H1*. Autistic individuals perform worse than comparison participants on TBPM, but not on EBPM independent of age (interaction between group and PM type).
*H2*. TBPM decreases more with age than EBPM, independent of autism (interaction between PM type and age).
*H3*. There will be a similar age effect in both autistic individuals and comparison participants.

Exploratively, we will assess whether autistic participants used different strategies compared to comparison participants when performing the task by exploring the number of times participants monitored time and whether ongoing task performance (time to complete the task and accurately sorted items) suffered during task performance. In addition, we will explore whether there is an age-PM paradox, as described in literature concerning typical aging, in autistic individuals. Furthermore, as PM is hypothesized to be essential for functioning in daily life, we will examine whether real-life cognitive complaints are related to PM task performance. Finally, as there appears to be a female advantage when it comes to PM (Hering et al., 2014; Palermo et al., 2016) independent of diagnosis, we will explore sex effects.

## Methods

### Participants

Participants (*n* = 193 (autistic *n* = 82 and non-autistic *n* = 111)) aged between 30 and 85 years were recruited via several clinical institutions across the Netherlands, (social) media advertisements in autism networks, and the social network of the researchers, research assistants, and students. A subset of participants took part in previous waves of an overarching ongoing study (Geurts et al., 2021). The following exclusion criteria were applied to all participants in this study: (a) intellectual disability and/or intelligence quotient (IQ) score below 70, (b) insufficient understanding of Dutch language, (c) a history of neurological disorders (e.g. epilepsy, stroke, multiple sclerosis), schizophrenia, or having experienced more than one psychosis, (d) Mini–Mental State Examination <18, and (e) current alcohol or drug dependency. For the comparison group, this was supplemented with (a) present/past diagnosis of an autism spectrum classification (ASC); (b) present/past diagnosis of an ASC, a total score higher than 32 on the Autism Quotient (AQ), or ASC in close family members (i.e. parent(s), child(ren), brother(s), sister(s)); (c) present/past diagnosis of attention deficit/hyperactivity disorder (ADHD) or a total score of six or higher on the ADHD-SR (self-report) subscales; or (d) a diagnosis of ADHD in close family members. Participants in the ASC group were required to (a) have a clinical *Diagnostic and Statistical Manual of Mental Disorders* (*DSM*; IV or 5) diagnosis of an ASC and (b) score above the cut-off on both the Autism Diagnostic Observation Schedule (ADOS)-2 (social affect >6 and/or total score >8) and the AQ >25.

### Transparency and openness

All analyses are preregistered before data collection was completed at AsPredicted (#34249). The data are made publicly available.

### Community involvement statement

In the current research, a stakeholder group consisting of four autistic older adults (two men, two women) was involved in all major aspects. In three to four meetings a year, the study design, materials, results, and dissemination of results are discussed. In this study, the group helped pilot the current task and were asked to pay special attention to feasibility of administering such a task to autistic adults. Interpretation of the results was also discussed. Their valuable feedback was incorporated.

### Materials

#### PM—Amsterdam Breakfast Task

The Amsterdam Breakfast Task is an adaptation of the Dresden Breakfast Task (also see Altgassen et al., 2012). The computer-based task (for graphical representation, see Figure S1) simulates a scenario in which the participant has to make breakfast before a friend arrives. The participant is asked to prepare six items (eggs, coffee, tea, croissants, hot milk, and toast) for breakfast by clicking on the correct item at the correct moment. These items are divided into three TBPM items and three EBPM items. The TBPM items require the participant to click on an item (oven, eggs, and teapot) after a certain amount of time has passed (2, 3, and 4 min, respectively). An item is correct if the item is clicked within a ±10-s window. The EBPM items require the participant to click on an item after an event takes place (boiling sound of the eggs, clicking sound of the toaster, and ringing of the doorbell to start the coffee machine). A task is considered to be correctly completed if the item is clicked within 10 s after the event occurred. Next to the PM items, the participant is asked to clear the dishes (ongoing activity; cutlery, plates, glasses) from the dishwasher to their appropriate place in the cupboard as marked on the screen. All items, including the ongoing activity task, need to be completed within 5 min. To keep track of the time, participants are able to click on a hidden clock which will appear for a few seconds when clicked on. The task automatically stops after 6 min.

Outcome variables of this task are accuracy on TBPM (0–3), EBPM (0–3), total number of clock clicks, and ongoing task performance (total number of correctly sorted tableware, and time to completion).

#### Naturalistic PM tasks

This study was part of a larger study in which participants were required to perform several computer and paper-and-pencil tasks. At the start of the study, the participant was asked to place a token in a basket after every computer task (seven in total). Furthermore, the participants were asked to indicate after an hour of testing that it is time for a break (yes/no). We registered the time that a participant arrives at the university for their testing appointment (difference in minutes between the agreed-upon time and the arrived time). Finally, the participants were asked to fill out a survey about their experience during the testing session within 24–36 h after their testing session. We registered whether participants filled out the questionnaire (on time (within 24–36 h)/early/late/not). This will lead to three TBPM items (on time appointment (in minutes), on time survey, break) and one EBPM item (tokens).

#### Everyday cognitive complaints—Cognitive Failures Questionnaire

The Cognitive Failures Questionnaire (CFQ) (Broadbent et al., 1982; Merckelbach et al., 1996) consists of 25 items about their cognitive complaints in daily life. The self-report questionnaire consists of everyday memory slips and lapses. Items are scored on a 5-point Likert-type scale (0–4), resulting in a total summary score between 0 and 100. The CFQ has excellent internal consistency in the current sample (*α* = 0.97).

### Procedure

Written informed consent was obtained from all participants. The study was approved by the ethical review board of the Department of Psychology of the University of Amsterdam (2018-BC-9285). This study was performed conforming the principles of the Helsinki Declaration of 1975, as revised in 2008. Participants underwent extensive phenotyping and neurocognitive testing (for a full description of procedures, see Geurts et al., 2021). Data used in this study are obtained during Wave 3 of a multi-cohort accelerated longitudinal study, and for all participants, it was the first time they were performing these tasks. Participants received compensation for travel and a small reward for participation.

### Statistical analyses

Frequentist analyses were run in R (RStudio Team, 2015), Bayesian analyses in JASP (JASP Team, 2020). We excluded those participants who have not fully understood the Amsterdam Breakfast Task, defined as participants who click all icons in the first 20 s. In addition, we excluded participants who did not perform the ongoing task. Outliers are not expected in the laboratory PM task. In the naturalistic task, we replaced outliers (i.e. scores 3 *SD* above the mean) with the next extreme value.

We performed a Task (Event/Time)*Group (Autism/Comparison)*Age (Young <50 years/Old >55 years) design to examine whether there is an age effect on task performance, and whether this effect is different in the two groups (Autism/Comparison) using a repeated measures analysis of variance (ANOVA). Separate 2 × 2 ANOVAs will be carried out to assess Task*Group and Task*Age interactions.

With the current sample (*n* = 193), we were able to detect small to medium effects (Cohens *d* = 0.4 when looking at two groups and *d* = 0.48 when looking at four groups).

We used the Benjamini–Hochberg correction to decrease the chance of a false discovery (based on five tests for the Amsterdam Breakfast Task, and five tests for the naturalistic task).

We explored whether autistic participants used different strategies compared to comparison participants when performing the task by exploring the number of times participants clicked on the animated clock, time to completion of the ongoing task, and accuracy of the ongoing task, by looking at Group (Autism/Comparison)*Age (Young/Old).

In addition, Bayes factors for the continuous lab-based and naturalistic task were calculated using ANOVA in JASP (JASP Team, 2020) with default priors. Frequentist analyses were supplemented with Bayesian statistics as we want to assess whether there is similar performance on PM between groups. BF_10_ expresses the probability of the data given H1 relative to H0, and BF_01_ expresses the probability of the data given H0 relative to H1 (please note that BF_01_ = 1/BF_10_). Bayes factors larger than 3 can be interpreted as substantial or stronger evidence for H1 (Lee & Wagenmakers, 2014).

Exploratively, we looked at the correlation between our lab-based task, more naturalistic PM tasks, reported cognitive complaints on the CFQ, and sex. To look whether there is a relationship between an ecological valid measure of cognitive complaints (i.e. total CFQ score), our lab-based PM task, and naturalistic PM, regression analyses were performed with cognitive complaints as a predictor and PM (either naturalistic or lab-based) as an outcome.

In addition to our preregistered analyses, we explored whether the PM tasks performed during the ongoing task showed group differences by performing a χ^2^ test between group and task to examine if different strategies were used in (older) autistic adults when performing the tasks. Moreover, we assessed whether there was an association between the number of clock clicks and performance on the Amsterdam Breakfast Task.

## Results

### Participant characteristics

Two comparison participants were excluded because they did not perform the ongoing task, and one comparison participant because they did not understand the task (clicked every icon within 20 s). As depicted in Table 1, no differences were found on age, IQ, education, or sex distribution between the groups, but as intended, the autism group had a higher AQ score.

**Table 1.** Participant characteristics.

	Autism (*n* = 82)		Comparison (*n* = 111)			Statistical value	*p* value	Cohens *d*
	M (*SD*)	Min–max	M (*SD*)	Min–max				
Age	54.48 (15.12)	30.68–85.13	55.41 (14.98)	30.45–85.33		*t* = −0.426	0.67	0.06
IQ	114.4 (16.21)	73–147	112.05 (17.02)	73–144		*t* = 0.978	0.33	0.14
AQ	34.8 (7.42)	14–48	13.24 (6.28)	2–30		*t* = 21.279	<0.001	3.14
Sex (*n*, % male)	56 (68%)	−	68 (61%)	–		χ^2^ = 0.73	0.39	
Education^a^	1/0/0/6/15/30/29		0/0/0/0/17/51/43			χ^2^ = 4.22	0.12	
Background (*n*, % non-Dutch)	4 (5%)		11 (10%)			χ^2^ = 1.04	0.31	
	Autism—Young (*n* = 48)	Autism—Old (*n* = 44)	Young vs old autism	Comparison young (*n* = 43)	Comparison old (*n* = 68)	Young vs old comparison	Statistical value	*p* value
	Mean (*SD*)	Mean (*SD*)	Cohens *d*	Mean (*SD*)	Mean (*SD*)	Cohens *d*		
Age	40.07 (6.16)	66.92 (7.5)	3.91	39.38 (6.66)	67.08 (7.52)	3.92	*F* = 228^b^	<0.001
IQ	109.68 (16.79)	118.48 (14.68)	0.55	105.35 (16.98)	117.02 (16.33)	.70	*F* = 6.64^c^	<0.001
AQ	36.03 (7.28)	33.75 (7.47)	0.31	11.74 (5.26)	14.82 (6.64)	.51	*F* = 156.46^d^	<0.001
Sex (*n*, % male)	32 (66.7)	24 (54.5)		27 (62.3)	38 (55.8)		χ^2^ = 1.5	0.68
Education^a^	0/0/0/0/7/14/17	1/0/0/6/8/16/12		0/0/0/0/7/20/16	0/0/0/0/10/25/26		χ^2^ = 7.67	0.27

IQ: intelligence quotient; AQ: Autism Quotient; ASC: autism spectrum classification.

For group comparisons, educational levels 1–5 were merged to prevent empty cells. Age young <50 years, age old > 55 years.

a Education ranges from 1 (primary education not finished) to 7 (university level degree based on the Verhage coding system (1964).

b Younger < older.

c Older > younger.

d ASC > comparisons.

### Group differences

**Figure 1.**
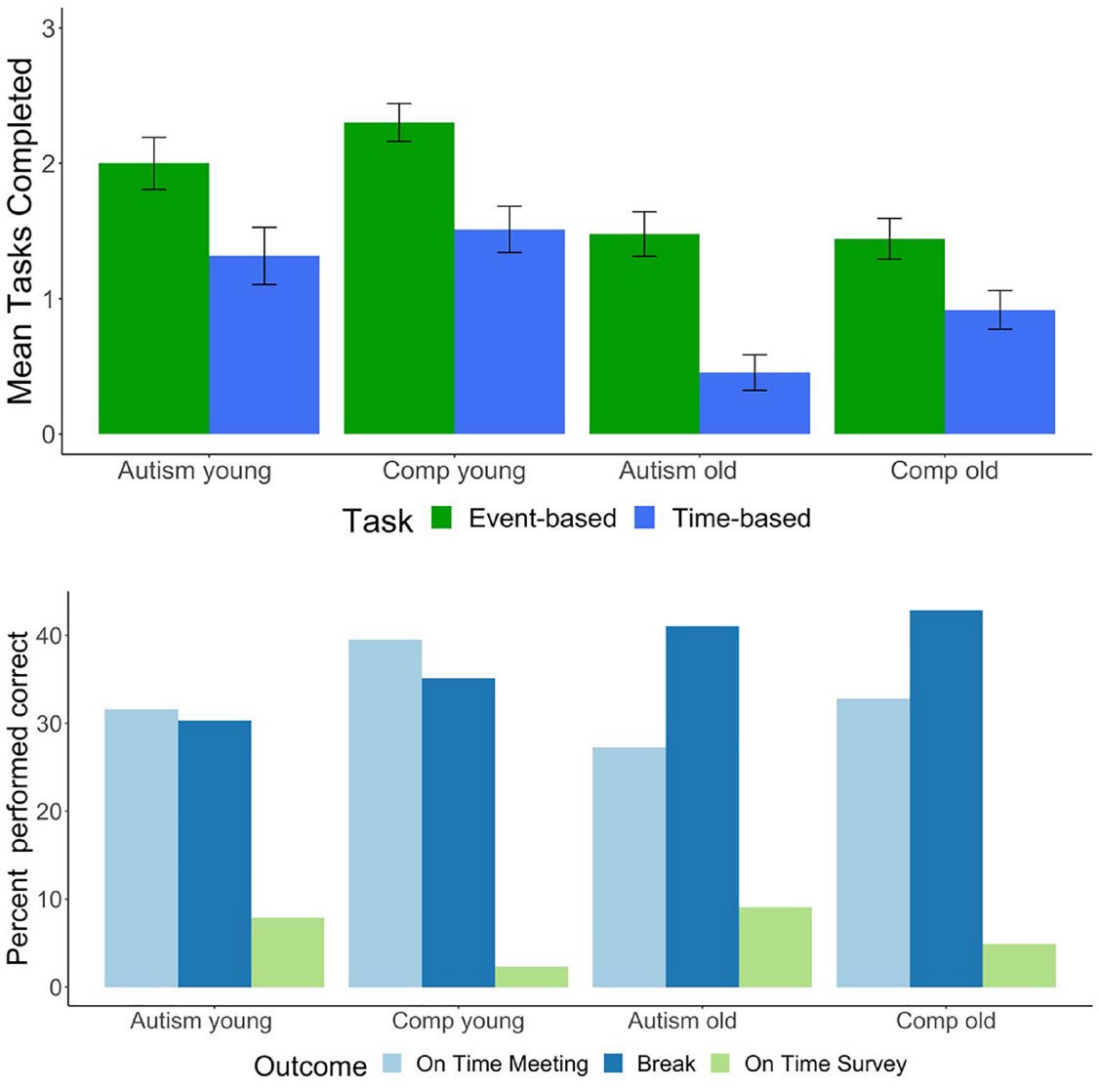
Mean scores per group on lab-based and naturalistic tasks. Error bars represent standard errors. Age young <50 years, age old >55 years. The top panel shows Amsterdam Breakfast Task; the lower panel shows naturalistic tasks. Comp: comparisons.

On the Amsterdam Breakfast Task, no Group*Task interaction was found (*F*(1, 191) = 2.14, *p* = 0.15), indicating that autistic and non-autistic adults performed similarly on both TBPM and EBPM tasks (see upper panel Figure 1). This finding is somewhat supported by Bayesian statistics (BF_10_ = 0.41). No group differences were found on any other indices of task performance. Furthermore, no group differences were found on any of the naturalistic tasks (see Table 2, and lower panel Figure 1 for results). Although this finding did not survive correction for multiple testing, more autistic individuals filled out the questionnaire after the testing session than comparisons (albeit not on time). Moreover, the Bayes factor (see Table 4) for the naturalistic task being on time with the survey and on the appointment shows moderate evidence that our data are more likely to be observed under the null hypothesis, that is, no difference between groups.

**Table 2.** Group outcomes on PM tasks.

	Performance Amsterdam Breakfast Task						
	Autism		Comparison		Statistical value	*p* value	Cohen’s *d*
	Mean (*SD*)	Min–max	Mean (*SD*)	Min–max			
Event based	1.72 (1.16)	0 to 3	1.81 (1.15)	0 to 3	*t* = −0.54	0.59	0.08
Time based	0.85 (1.17)	0 to 3	1.18 (1.15)	0 to 3	*t* = −1.93	0.06	0.28
Ongoing task performance (#correct)	28.46 (4.1)	10 to 30	28.8 (3.27)	10 to 30	*t* = −0.62	0.54	0.03
Ongoing task performance (time in seconds)	136.67 (65.31)	61 to 359	128.57 (57.43)	58 to 285	*t* = 0.90	0.37	0.13
Clock clicks	14.67 (9.78)	1 to 48	16.89 (11.81)	0 to 57	*t* = −1.43	0.16	0.20
Performance naturalistic tasks							
Tokens	7 (4.62)	0 to 28	8.46 (4.83)	0 to 28	*F* = 4.35	0.04	0.30
Appointment (in minutes)	11.8(14.1)	−20 to 60	8.32 (14.1)	−42.72 to 60	*F* = 2.70	0.10	0.24
Questionnaire (in hours)	5.52 (144.24)	−583.38 to 377.54^a^	26.12 (78.66)	−261 to 285	*F* = 0.89	0.35	0.17
Questionnaire: *n* (%) on time	7 (8.5)	–	4 (3.6)	–	χ^2^ = 9.88	0.02	NA
Questionnaire: *n* (%) non-responders	24 (29.3)	–	55 (49.5)	–			NA
Break *n* (%) yes	26 (36.1)	–	38 (38.4)	–	χ^2^ = 0.09	0.76	NA

PM: prospective memory; NA: not applicable.

Age younger <50 years, age older >55 years.

a Some participants filled out our survey at the moment they got the invitation for the test appointment causing negative numbers.

As most individuals completed the ongoing task before the end of the Amsterdam Breakfast Task and thus while still working on (some of) the PM tasks, we explored whether there were group differences for those PM tasks performed during the ongoing task, but none were found (hot milk (EBPM) (χ^2^ = 0.12, *p* = 0.72), croissant (TBPM) (χ^2^ = 2.65, *p* = 0.10)).

### Age effects

Repeated measures ANOVA showed that older individuals performed worse on both EBPM and TBPM as measured with the Amsterdam Breakfast Task (*F*(1, 184) = 24.47, *p* < 0.001)), but no Age*Task (*F*(1, 184) = 0.002, *p* = 0.96) or Group*Age*Task (*F*(1, 182) = 3.36, *p* = 0.07) effects were found. Bayesian statistics (BF_10_ = 0.17) for the effect of Age*Task show that there is strong evidence that our data are more likely to be observed under the null hypothesis. The Bayes factor for Group*Age*Task (BF_10_ = 1) did not allow us to make a definite conclusion about our hypothesis. It appears that older individuals made less clock clicks than younger individuals, but this was similar in both autism and comparisons. A higher number of clock clicks was significantly associated with better performance on both EBPM tasks (*t* = 5.86, *p* < 0.001, *r*^2^_adj_ = 0.15) and TBPM tasks (*t* = 6.22, *p* < 0.001, *r*^2^_adj_ = 0.16). Moreover, a Group*Age interaction was found on time to completion of the ongoing task (also see Table 3). Older autistic individuals were slower in completing the ongoing tasks than younger autistic individuals and older comparisons, but not younger comparisons. Older comparisons were significantly faster than older autistic individuals, but not than both younger groups. Younger comparisons and autistic individuals did not differ from each other. Time to complete the ongoing task was not associated with task performance on EBPM (*t* = −0.22, *p* = 0.83, *r*^2^_adj_ = −0.004) or TBPM (*t* = 0.79, *p* = 0.43, *r*^2^_adj_ = −0.002).

**Table 3.** Age by group outcomes on PM tasks.

Mean (*SD*)	Performance Amsterdam Breakfast Task per age group					
	Autism—Young	Autism—Old	Comparison young	Comparison old	Age	Age*Group
	Mean (*SD*)	Mean (*SD*)	Mean (*SD*)			
Event based	2 (1.19)	1.48 (1.09)	2.3 (0.91)	1.44 (1.18)	***F*** **(1, 184)** **=** **18.67, ***p***** **<** **0.001**	*F*(1, 182) = 1.05, *p* = 0.31
Time based	1.32 (1.3)	0.45 (0.87)	1.51 (1.12)	0.92 (1.11)	***F*** **(1, 184)** **=** **17.82, *p*** **<** **0.001**	*F*(1, 182) = 0.66, *p* = 0.42
Ongoing task performance (#correct)	28.5 (4.37)	28.43 (3.91)	29.49 (0.88)	28.21 (4.27)	*F*(1, 182) = 1.81, *p* = 0.18	*F*(1, 182) = 1.20, *p* = 0.28
Ongoing task completion (in seconds)	116.11 (51.11)	154.43 (71.35)	138.35 (66.59)	121.46 (48.49)	*F*(1, 182) = 0.78, *p* = 0.38	***F* **(1, 182)** **=** **9.73,** *p* **=** **0.002****
Clock clicks	17.05 (10.27)	12.61 (8.94)	20.74 (11.16)	14.28 (12.1)	***F* **(1, 182)** **=** **12,** *p* **<** **0.001****	*F*(1, 182) = 0.39, *p* = 0.53
Performance naturalistic tasks						
Tokens	6.92 (3.86)	7.07 (5.24)	9 (4.46)	8.12 (5.28)	*F*(1, 176) = 0.31, *p* = 0.58	*F*(1, 176) = 0.50, *p* = 0.48
Appointment	9.66 (14.85)	13.61 (13.3)	5.64 (10.96)	9.68 (17.02)	*F*(1, 182) = 3.48, *p* = 0.06	*F*(1, 182) = 00, *p* = 0.98
Questionnaire (in hours)	−0.65 (186.59)	10.53 (100.59)	26.57 (77.02)	27.43 (84.69)	*F*(1, 107) = 0.08, *p* = 0.78	*F*(1, 107) = 0.05, *p* = 0.82
Questionnaire: *n* (%) on time	3 (7.9)	4 (9.1)	1 (2.3)	3 (4.9)	χ^2^ = 1.20, *p* = 0.75	χ^2^ = 10.58, *p* = 0.31
Questionnaire: *n* (%) non-responders	12 (31.6)	12 (27.3)	20 (46.5)	31 (50.8)		
Break *n*(%)	10 (30.3)	16 (41)	13 (35.1)	24 (42.9)	χ^2^ = 1.46, *p* = 0.23	χ^2^ = 1.67, *p* = 0.64

PM: prospective memory.

Age younger <50 years, age older >55 years.

Bold figures are significant after Benjamini–Hochberg correction.

Concerning naturalistic tasks, we did not find any differences in age (younger vs older) or Age*Group interactions (see Table 3). Most Bayes factors confirm that the data concerning these naturalistic tasks are more likely to occur under the null hypothesis (no difference between age groups, and no differential effects of age between autism and comparisons). Bayes factors for age effects on being on time for the test appointment did not allow us to draw conclusions (also see Table 4).

**Table 4.** Bayesian statistics of performance on PM tasks.

	Bayesian statistics Performance Amsterdam Breakfast Task					
	Group (Autism/Comparison)		Age (younger/older)		Age*Group	
	BF^10^	BF^01^	BF^10^	BF^01^	BF^10^	BF^01^
Event based	**0.18**	**5.50**	**711.41**	**0.001**	**0.30**	**3.31**
Time based	0.90	1.12	**491.82**	**0.002**	**0.18**	**5.60**
Ongoing task performance (#correct)	**0.19**	**5.24**	0.36	2.79	0.39	2.60
Ongoing task performance (in seconds)	**0.24**	**4.22**	**0.22**	**4.57**	**15.39**	**0.07**
Clock clicks (#)	0.39	2.56	**29.53**	**0.03**	**0.27**	**3.77**
Performance naturalistic tasks						
Tokens (#)	1.18	0.85	**0.18**	**5.56**	**0.28**	**3.58**
On time appointment (in seconds)	0.56	1.80	0.68	1.46	**0.22**	**4.46**
On time questionnaire (in hours)	**0.30**	**3.38**	**0.21**	**4.76**	**0.3**	**3.33**

PM: prospective memory.

Bold figures indicate substantial or stronger evidence for either hypothesis: BF^10^ expresses the probability of the data given H1 relative to H0, and BF^01^ expresses the probability of the data given H0 relative to H1.

### Exploratory analyses

More cognitive complaints (on the CFQ) were associated with a decrease in performance on TBPM, but the amount of explained variance was low (*r*^2^ = 0.03). The relation between cognitive complaints and TBPM was not affected by age, group, or the interaction Age*Group. Moreover, none of the other outcomes (TBPM, being on time and number of tokens) were significantly associated with cognitive complaints (also see Table S2). In addition, no effects of Sex, Sex*Group, Sex*Age, or Sex*Age*Group were found on lab-based or naturalistic PM performance (see Table S3).

### Sensitivity analyses

Sensitivity analyses were performed to test the robustness of our results. First, as some of the outcome variables can be seen as “count” data, and do not necessarily meet the assumption of normality, all analyses were repeated with non-parametric statistics. The results of these tests mimic those described above (for full results, see Table S1). Second, while there was no significant difference between autistic and non-autistic adults in IQ, older adults in both groups had a higher IQ than younger adults. All analyses on age effects were rerun with a sample matched on IQ. The results of these analyses also mimic the results of our main analyses (for full results, see Table S5).

## Discussion

We aimed to examine aging patterns of PM in autistic adults to assess whether aging affects TBPM and EBPM equally in autistic individuals compared to typical aging. In contrast to our hypothesis, we found no group differences on either lab-based task performance (TBPM or EBPM) or naturalistic PM tasks. Importantly, our data more likely indicate equal performance in autistic adults and non-autistic adults. Also contrasting our hypothesis, TBPM and EBPM showed a similar age-related difference. In concurrence with our hypothesis, we found evidence for a parallel developmental trajectory of PM in autistic and non-autistic individuals. Our results show no effect of age on naturalistic tasks, but age did affect our lab-based measure. This supports the age paradox often described in PM research and we show that this paradox is also observed in autistic people.

In contrast to previous work (Landsiedel et al., 2017; Sheppard et al., 2018), we did not find a difference between the autism and comparison group on either EBPM or TBPM. Bayesian statistics even suggested that our data likely pointed to equal performance in both tasks in both groups. According to the multi-process framework (Einstein & McDaniel, 1996; McDaniel & Einstein, 2000), with higher attentional load and cognitive demands, tasks will be more difficult to correctly perform by those with reduced cognitive resources. The ongoing task in the Amsterdam Breakfast Task was relatively simple (although no ceiling effects were seen in performance), designed to be ecologically valid and was expected to have low cognitive demands. Moreover, many people did not need the entire duration of the task to finish the ongoing task. Exploratively, we examined whether group performance was different during the ongoing task, but this was not the case. This could be an indication that PM functions similarly in autistic and non-autistic adults when surroundings (e.g. an ongoing task) have low cognitive demands. Research has indicated that autistic individuals do perform differently on more classical lab-based tasks with ongoing tasks that have higher cognitive demands (see Altgassen & Koch, 2014; Williams et al., 2014). However, we currently do not know whether this translates into difficulties in daily life.

With increasing age and decreasing cognitive resources, cognitive demands tend to become more difficult to meet, leading to a decline in performance (Salthouse, 2004). While we indeed found that older individuals completed fewer tasks correctly (both EBPM and TBPM) on the lab-based Amsterdam Breakfast Task than younger individuals, both age groups did not differ on the naturalistic tasks. This phenomenon has often been described as the age paradox of PM (Henry et al., 2004; Niedźwieńska & Barzykowski, 2012; Rendell & Craik, 2000). While meta-analytic evidence suggests (Henry et al., 2004) that older individuals show superior performance in naturalistic tasks, in line with more recent studies (Kvavilashvili et al., 2013; Schnitzspahn et al., 2020), our data point to equal performance on naturalistic tasks in both age groups. While it could be argued that our tasks were relatively easy, all tasks show enough variation to assume that they were not equally easy for all participants. It could however be, that because participants have experience with the naturalistic tasks, these tasks were generally easier to perform. Moreover, it was allowed to use reminders to perform the naturalistic tasks that were performed outside the lab. This leaves the possibility that younger participants rely more on their PM to perform these tasks, while older individuals rely more on the mechanisms they use to help prevent PM fails. Although it is currently unclear how naturalistic PM develops longitudinally, we are the first to show this pattern (i.e. decrease in performance on lab-based tasks, and equal performance on naturalistic tasks) in autistic individuals. Thus, it appears that PM in autistic individuals shows a pattern of parallel aging, as is also found in other cognitive functions (Davids et al., 2016; Lever & Geurts, 2016; Torenvliet et al., 2022, 2023; Tse et al., 2019). However, longitudinal studies are warranted to assess this effect over time.

Although many explanations have been given for the age paradox of PM, the age paradox could also reflect the reliability and validity of the current measures used to assess PM, both in, and outside the lab. Lab-based tasks have their merits; they allow us the ability to control the environment, and make manipulations to it, thus providing us with the opportunity to draw conclusions about possible processes underlying cognitive functioning. However, with controlling and manipulating the environment, ecological validity of lab-based measures is drastically reduced (Holleman et al., 2020; Nastase et al., 2020; van Atteveldt et al., 2018). While one could argue that this emphasizes the need to include both naturalistic and experimental tasks, it also raises questions whether we are actually measuring the same cognitive function in the lab as in the real world. It is often suggested that PM is an essential function for daily living (Hering et al., 2018; Sheppard et al., 2020), and we did find a correlation between real-life cognitive complaints and performance on TBPM, although this association was very low (*r* = 0.03), and the internal consistency of our naturalistic tasks was low (α = 0.1). Moreover, this is also reflected in the very low correlations (*r* between −0.13 and 0.001) between our lab-based and naturalistic tasks (full table available, Supplemental Table S4). Construct validity is problematic if there is no “gold standard.” While we feel that our measures at least have face validity, the field should focus on the development of valid and reliable measures.

While our results indicate equal performance of autistic and non-autistic adults on the Amsterdam Breakfast Task, and we did not find any differential age effects between the groups, our results do indicate a possible difference in strategy (Livingston & Happé, 2017). Age effects were found in time to complete the ongoing task, where we found that older autistic individuals took more time in completing the ongoing task compared to younger autistic individuals and older comparisons. This could indicate that older autistic adults allocated attentional resources needed for performing a dual task in a different way than the other groups. However, we should interpret this with caution as time to complete the ongoing task was not associated with task performance. Regardless of diagnostic classification, we found that older adults relied less on the clock in completing the task (reflected in less clock clicks) than younger adults. Moreover, a higher number of clock clicks was associated with better performance in both TBPM and EBPM, indicating a benefit of looking at the time. While it appears surprising that EBPM performance was also associated with the number of clock clicks, this might reflect overall monitoring behavior resulting in better screening of the environment, or general higher engagement in the task. This could indicate that people are using compensation strategies to adapt their behavior up to an optimal performance.

This study has several strengths worth mentioning. First, we are the first to perform both naturalistic and lab-based PM tasks in the same large sample of autistic and non-autistic (older) adults. Second, due to our use of Bayesian statistics in addition to standard frequentist statistics, we were able to draw conclusions about null findings, and we show that it is most likely that autistic and non-autistic adults do not differ on PM and show a similar age pattern. Nevertheless, the current findings should be viewed in the light of some weaknesses. Previous research has suggested differential age effects on naturalistic EBPM versus TBPM tasks (Kvavilashvili et al., 2013; Niedźwieńska & Barzykowski, 2012; Schnitzspahn et al., 2020). EBPM naturalistic tasks are difficult to design (Phillips et al., 2008), and our EBPM naturalistic task (putting tokens in a basket after each computer task) is arguably more of a hybrid between lab-based and naturalistic PM task. It could even be argued that it might even be more of an activity-based PM task, and thus easier than EBPM. Moreover, during our naturalistic TBPM task, we cannot be sure that every person received the same amount of reminders before the testing appointment, although we do know that every person received at least two. Finally, it could be argued that the cognitive demands our ongoing task puts on the participants were not comparable to those of classical lab-based task to measure PM. However, cleaning out the dishwasher (albeit virtually) is arguably a more ecological valid task than visuospatial working memory tasks, or word organization tasks. Nevertheless, it could be that performance on the Amsterdam Breakfast Task was better than on comparable tasks due to lower cognitive demands of the ongoing task.

In conclusion, our results point to parallel development of PM in autistic and non-autistic adults. Moreover, the often-described age paradox is also found in autistic individuals. However, future research is needed to test whether this paradoxical development over age of naturalistically measured PM and experimentally measured PM might reflect that current experimental measures do not capture real-life PM.

## Supplemental Material

sj-docx-1-aut-10.1177_13623613231225489 – Supplemental material for Remembering the future; prospective memory across the autistic adult’s life span Supplemental material, sj-docx-1-aut-10.1177_13623613231225489 for Remembering the future; prospective memory across the autistic adult’s life span by Annabeth P Groenman, Carolien Torenvliet, Tulsi A Radhoe, Joost A Agelink van Rentergem, Wikke van der Putten, Mareike Altgassen and Hilde M Geurts in Autism
