# Supplementary material for: Remembering the future; prospective memory across the autistic adult’s life span
Source: Autism. 2024 Jan 19;28(9):2254–66. doi: 10.1177/13623613231225489 (PMC11403918; doi:10.1177/13623613231225489)
Supplement: sj-docx-1-aut-10.1177_13623613231225489 – Supplemental material for Remembering the future; prospective memory across the autistic adult’s life span [file sj-docx-1-aut-10.1177_13623613231225489.docx]

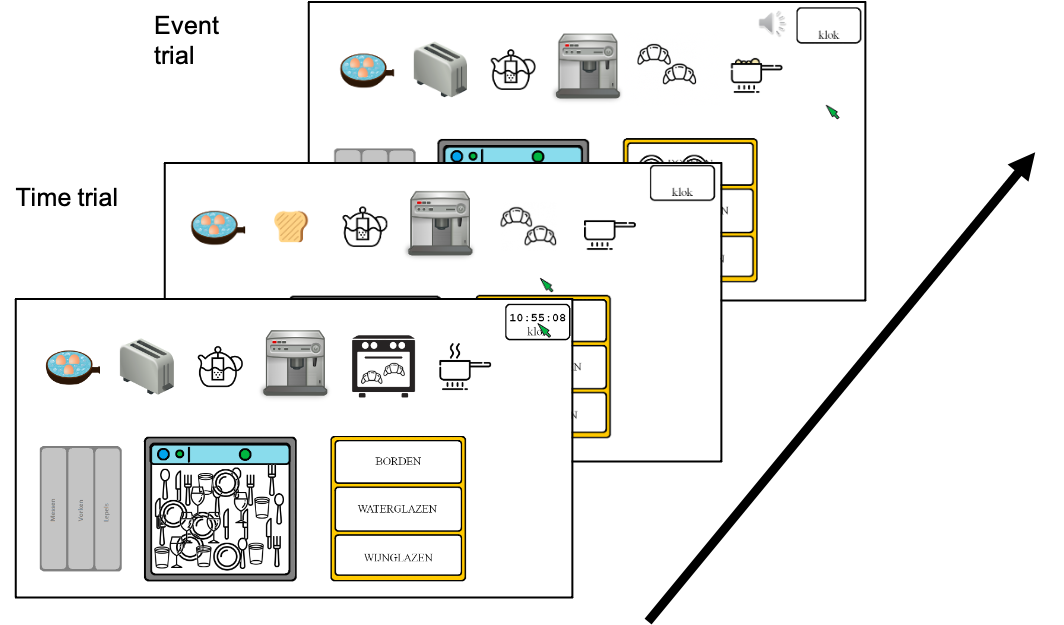


Figure S1. Graphical description of the Amsterdam Breakfast Task.

Table S1 Nonparametric tests of prospective memory outcomes.

|  |  | Performance Amsterdam breakfast task per age group | | | | | | |
| --- | --- | --- | --- | --- | --- | --- | --- | --- |
|  | Autism- young | Autism- old | | Comparison young | Comparison Old | Age | Group | Group4 |
|  | Mean (sd) | Mean (sd) | | Mean (sd) | Mean (sd) | P value | P value | P value |
| Event based | 2 (1.19) | 1.48 (1.09) | | 2.3 (0.91) | 1.44 (1.18) | **<0.001** | 0.60 | **<0.001*** |
| Time based | 1.32 (1.3) | 0.45 (0.87) | | 1.51 (1.12) | 0.92 (1.11) | **<0.001** | 0.03 | **<0.001*** |
| Ongoing task performance (#correct) | 28.5 (4.37) | 28.43 (3.91) | | 29.49 (0.88) | 28.21 (4.27) | 0.06 | 0.78 | 0.19 |
| Ongoing task completion (in sec) | 116.11 (51.11) | 154.43 (71.35) | | 138.35 (66.59) | 121.46 (48.49) | 0.55 | 0.42 | 0.07 |
| Clock clicks | 17.05 (10.27) | 12.61 (8.94) | | 20.74 (11.16) | 14.28 (12.1) | **<0.001** | 0.28 | **<0.001** |
|  |  |  | Performance naturalistic tasks | | | | | |
| Tokens | 6.92 (3.86) | 7.07 (5.24) | | 9 (4.46) | 8.12 (5.28) | 0.94 | **0.01** | 0.08 |
| On time Appointment | 9.66 (14.85) | 13.61 (13.3) | | 5.64 (10.96) | 9.68 (17.02) | 0.10 | 0.09 | 0.08 |
| On time Questionnaire (in hours) | -0.65 (186.59) | 10.53 (100.59) | | 26.57 (77.02) | 27.43 (84.69) | 0.65 | 0.36 | 0.67 |

Age <50/>55, group= ASC/COM, group4= ASC young, ASC old, COM young, COM old. *both young groups differed from both old groups.

Supplementary info on cognitive complaints analyses and sex effects.

Table S2. Cognitive complaints predicting

|  | Main effect CFQ | | Group*CFQ | | Age*CFQ | | Age*Group*CFQ | |
| --- | --- | --- | --- | --- | --- | --- | --- | --- |
|  | b | p | b | p | b | p | b | p |
| Event based | -0.01 | 0.12 | 0.001 | 0.91 | -0.01 | 0.25 | 0.01 | 0.65 |
| Time based | **-0.01** | **0.005** | -0.01 | 0.61 | -0.01 | 0.55 | -0.03 | 0.22 |
|  |  |  |  |  |  |  |  |  |
| Tokens | -0.03 | 0.02 | 0.04 | 0.33 | -0.02 | 0.68 | 0.01 | 0.94 |
| On time Appointment | 0.02 | 0.65 | -0.02 | 0.87 | -0.07 | 0.58 | 0.44 | 0.18 |
| On time Questionnaire | -0.22 | 0.56 | -1.92 | 0.09 | -0.07 | 0.96 | 0.96 | 0.77 |

^Note. reference age is young, reference group is comparisons^

Table S3. Sex effects on prospective memory

|  | Sex | | Group*sex | | Age*sex | | Age*Group*sex | |
| --- | --- | --- | --- | --- | --- | --- | --- | --- |
|  | F | p | F | p | F | p | F | p |
| Event based | 1.30 | 0.26 | 0.06 | 0.81 | 0.06 | 0.81 | 2.45 | 0.12 |
| Time based | 1.39 | 0.24 | 0.46 | 0.50 | 0.46 | 0.50 | 0.00 | 0.99 |
|  |  |  |  |  |  |  |  |  |
| Tokens | 2.21 | 0.14 | 0.05 | 0.81 | 0.05 | 0.81 | 1.02 | 0.31 |
| On time Appointment | 0.35 | 0.55 | 0.90 | 0.34 | 0.90 | 0.34 | 0.50 | 0.48 |
| On time Questionnaire | 0.05 | 0.82 | 2.39 | 0.13 | 2.39 | 0.13 | 3.70 | 0.06 |

^note. reference age is young, reference group is comparisons, reference sex is male^

Table S4. Correlation matrix between lab based and naturalistic PM tasks.

|  | AOT EBPM | AOT TBPM | Tokens | On time Appointment in minutes | On time Survey |
| --- | --- | --- | --- | --- | --- |
| AOT EBPM | 1 | .54*** | -.01 | -.02 | .001 |
| AOT TBPM |  | 1 | .08 | -.13 | -.09 |

*p<.05, **p<.01, ***p<.001

Table S5. Analyses on age effect with IQ matched groups

|  | | Performance Amsterdam Breakfast Task per age group | | | | | |
| --- | --- | --- | --- | --- | --- | --- | --- |
|  | Autism - Young | | Autism - Old | Comparison Young | Comparison Old | Age | Age*group |
|  | Mean (sd) | | Mean (sd) | Mean (sd) | Mean (sd) |  |  |
| Event based | 1.97 (1.22) | | 1.42 (1.03) | 2.44 (0.84) | 1.31 (1.2) | ***F*(1,144)=** **23,12, *p*<.001** | *F*(1,144)=2.65, *p* =.11 |
| Time based | 1.32 (1.32) | | 0.42 (0.83) | 1.61 (1.1) | 0.78 (1.04) | ***F*(1,144)= 23.23, *p*<.001** | *F*(1,144)=.03, *p* =.85 |
| Ongoing task performance (#correct) | 28.88 (3.43) | | 28.06 (4.45) | 29.53 (0.84) | 27.78 (4.85) | *F*(1,144)= 4.47, *p* =.04 | F(1,144)= 0.54, *p* =.46 |
| Ongoing task completion (in sec) | 116.97 (53.59) | | 163.67 (76.55) | 146.25 (66.98) | 116.6 (43.04) | *F*(1,144)= .27, *p* =.60 | **F(1,144)= 14.69, *p* <.001** |
| Clock clicks | 17.26 (10.36) | | 11.67 (9.07) | 20.86 (9.12) | 12.44 (11.47) | ***F*(1,144)= 18.09**, ***p* <.001** | F(1,144)=.70, *p*=.10 |
|  | | Performance naturalistic tasks | | | | | |
| Tokens | 6.38 (3.29) | | 7.06 (5.86) | 9.26 (4.75) | 8.33 (5.83) | *F*(1,139)= .04, *p* =.85 | F(1,139)= .89, *p*=.35 |
| Appointment | 9.18 (14.48) | | 11.97 (11.13) | 4.93 (10.57) | 10.89 (17.57) | *F*(1,144)= 3.80, *p* =.05 | F(1,144)= .46, *p*=.50 |
| Questionnaire (in hours) | -7.26 (215.07) | | 0.71 (111.86) | 18.9 (68.17) | 37.86 (65.41) | *F*(1,84)= .21, *p*=.64 | F(1,84)= .04, *p*=.85 |
| questionnaire: n(%) on time | 3 (8.8) | | 3(9.1) | 3 (2.8) | 3(6.7) | 𝜒^2^=1.31, *p*=.72 | 𝜒^2^=9, *p*=.44 |
| questionnaire: n(%) non responders | 11 (32.4) | | 9 (27.3) | 16 (44.4 | 24(53.3) |  |  |
| Break n(%) | 9 (30) | | 12 (38.7) | 12 (36.4) | 16 (32.7) | 𝜒^2^=0.35, *p*=.84 | 𝜒^2^=3.55, *p*=73.70 |
